# Clinical Characteristics and Outcomes of Fungal Keratitis in the United Kingdom 2011–2020: A 10-Year Study

**DOI:** 10.3390/jof7110966

**Published:** 2021-11-12

**Authors:** Darren Shu Jeng Ting, Mohamed Galal, Bina Kulkarni, Mohamed S. Elalfy, Damian Lake, Samer Hamada, Dalia G. Said, Harminder S. Dua

**Affiliations:** 1 Academic Ophthalmology, Division of Clinical Neuroscience, School of Medicine, University of Nottingham, Nottingham NG7 2RD, UK; daliagsaid@gmail.com (D.G.S.); profdua@gmail.com (H.S.D.); 2 Queen’s Medical Centre, Nottingham NG7 2RD, UK; bina.kulkarni@nuh.nhs.uk; 3 Queen Victoria Hospital, East Grinstead RH19 3DZ, UK; mohamed.galal@nhs.net (M.G.); m.elalfy@nhs.net (M.S.E.); damian.lake@nhs.net (D.L.); s.hamada@nhs.net (S.H.); 4 Research Institute of Ophthalmology, Giza 12557, Egypt

**Keywords:** *Candida*, corneal infection, corneal ulcer, contact lens, fungal infection, *Fusarium*, infectious keratitis, keratoplasty

## Abstract

Fungal keratitis (FK) is a serious ocular infection that often poses significant diagnostic and therapeutic dilemmas. This study aimed to examine the causes, clinical characteristics, outcomes, and prognostic factors of FK in the UK. All culture-positive and culture-negative presumed FK (with complete data) that presented to Queen’s Medical Centre, Nottingham, and the Queen Victoria Hospital, East Grinstead, between 2011 and 2020 were included. We included 117 patients (*n* = 117 eyes) with FK in this study. The mean age was 59.0 ± 19.6 years (range, 4–92 years) and 51.3% of patients were female. Fifty-three fungal isolates were identified from 52 (44.4%) culture-positive cases, with *Candida* spp. (33, 62.3%), *Fusarium* spp. (9, 17.0%), and *Aspergillus* spp. (5, 9.4%) being the most common organisms. Ocular surface disease (60, 51.3%), prior corneal surgery (44, 37.6%), and systemic immunosuppression (42, 35.9%) were the three most common risk factors. Hospitalisation for intensive treatment was required for 95 (81.2%) patients, with a duration of 18.9 ± 16.3 days. Sixty-six (56.4%) patients required additional surgical interventions for eradicating the infection. Emergency therapeutic/tectonic keratoplasty was performed in 29 (24.8%) cases, though 13 (44.8%) of them failed at final follow-up. The final corrected-distance-visual-acuity (CDVA) was 1.67 ± 1.08 logMAR. Multivariable logistic regression analyses demonstrated increased age, large infiltrate size (>3 mm), and poor presenting CDVA (<1.0 logMAR) as significant negative predictive factors for poor visual outcome (CDVA of <1.0 logMAR) and poor corneal healing (>60 days of healing time or occurrence of corneal perforation requiring emergency keratoplasty; all *p* < 0.05). In conclusion, FK represents a difficult-to-treat ocular infection that often results in poor visual outcomes, with a high need for surgical interventions. Innovative treatment strategies are urgently required to tackle this unmet need.

## 1. Introduction

Infectious keratitis (IK) represents the leading cause of corneal blindness globally, with an estimated incidence of 2.5–799 cases per 100,000 population/year [1,2,3]. Subject to geographical, temporal and seasonal variations, bacteria and fungi are the most common causative organisms for IK, while viral and parasitic infections are less commonly reported [3,4,5,6,7,8,9]. The variations in the incidence and causes are mainly attributed to an underlying discrepancy in the risk factors (particularly contact lens wear, trauma and ocular surface disease), climate, access to a healthcare system, personal and environmental hygiene, and level of education [1].

Fungal keratitis (FK) often poses significant diagnostic and therapeutic dilemmas. It is most commonly observed in tropical/subtropical countries and regions with prevalent agricultural activity, accounting for 23–63% of all IK cases in these regions [1,10,11]. Compared to bacterial keratitis, FK is more frequently associated with guarded visual prognosis, primarily caused by the significant diagnostic challenge (due to low and slow culture yield), the propensity to deeper infection affecting the posterior cornea, limited antifungal treatment option, and resistance to treatment [4,12]. In addition, many cases of FK usually require therapeutic keratoplasty to achieve complete resolution of the disease, with many of them affected by the recurrence of infection or uncontrolled infection progressing to endophthalmitis and eventuating in evisceration/enucleation [12,13,14,15,16].

To date, the majority of the FK studies reported in the literature were conducted in developing or tropical/subtropical regions, including India, China and Nepal, where FK is more prevalent [4,17,18,19,20,21,22,23]. However, the results of those studies may not be readily applicable to populations in developed or temperate regions as the population characteristics, risk factors, underlying causes and management of FK can vary significantly [1]. So far, there was only one large study that had specifically examined the outcome of FK in the United Kingdom (UK) in the past decade [24]. In view of the paucity of the literature and the clinical significance of the disease, this study aimed to examine the clinical characteristics, risk factors, outcomes, and prognostic factors of FK in the UK.

## 2. Materials and Methods

This was a retrospective study of all cases of FK that presented to two of the tertiary ophthalmic referral centres in the UK, namely the Queen’s Medical Centre, Nottingham, and the Queen Victoria Hospital, East Grinstead, between January 2011 and December 2020 (a 10-year period). The study was approved as a clinical audit by the Clinical Governance team in both Nottingham University Hospitals NHS Trust (Ref: 19-265C) and Queen Victoria Hospital NHS Foundation Trust (Ref: 21-539).

### 2.1. Case Identification and Definition

Potential cases of FK were first identified via the local microbiological database and hospital pharmacy database (based on the use of a topical antifungal treatment). Subsequently, the medical case records were examined to confirm the eligibility of the potential cases prior to inclusion into the study. In anticipation of the low prevalence of FK in the UK, both culture-positive and culture-negative presumed FK cases were included in this study. Culture-positive FK was defined as the presence of clinical FK with confirmation of the causative fungal pathogen on microbiological culture. Culture-negative presumed FK was diagnosed based on the presence of typical clinical findings (see below) and/or suggestive clinical course such as non-improvement/deterioration with intensive topical antibiotic treatment alone, which subsequently required intensive topical antifungal treatment to improve and resolve the infection. Co-infection of FK with culture-positive bacterial keratitis cases were included but pure bacterial keratitis cases were excluded from this study. Identification of fungal and bacterial infection was primarily based on conventional culture morphologies. Other types of infection, including viral and parasitic keratitis, were also excluded from this study.

### 2.2. Data Collection

**Figure 1:**
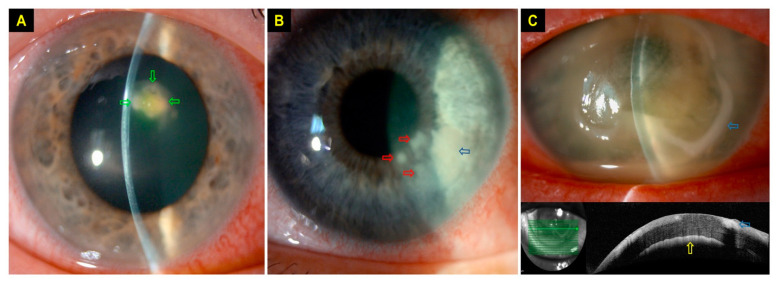
Slit-lamp photography demonstrating typical signs of fungal keratitis (FK). (**A**) A small contact lens (CL)-related FK with multifocal infiltrates (green arrows) with feathery borders. (**B**) A CL-related FK with multifocal infiltrates of feathery borders, with an area of main infiltrate (blue arrow) and three satellites lesions (red arrows). (**C**) A case of severe FK with hypopyon, ring infiltrate (blue arrows) and endothelial plaque (yellow arrow).

Relevant data, including demographic factors, risk factors, clinical characteristics, types of fungi, corrected-distance-visual-acuity (CDVA), pre-existing ocular co-morbidities that could affect the visual prognosis, management, outcome and complications, were collected using a standardised Microsoft Excel proforma. Risk factors were divided into (1) contact lens wear; (2) trauma; (3) ocular surface diseases (e.g., dry eye, meibomian gland dysfunction, neurotrophic keratopathy, previous corneal infection, recurrent corneal erosion syndrome, limbal stem cell deficiency, cicatricial conjunctivitis, band keratopathy, and bullous keratopathy); (4) lid diseases (e.g., entropion, ectropion, distichiasis/trichiasis, and exposure keratopathy); (5) use of topical corticosteroids; (6) previous/recent history of corneal surgery (e.g., corneal graft, pterygium surgery, corneal collagen cross-linking and corneal debridement/delamination), and (7) systemic immunosuppression (e.g., diabetes, systemic immunosuppressive treatment, malnutrition, and immunodeficiency). Slit-lamp photographs and anterior segment optical coherence tomography (AS-OCT) were examined for the presence of any typical characteristics of FK, including the feathery border of the infiltrate, ring infiltrate, satellite lesions (small infiltrates near the main infiltrative lesion), multifocal lesions (≥2 infiltrates either close or far apart from each other), and deep stromal/endothelial plaque (Figure 1A–C). Deep stromal infection was defined as the involvement of the posterior 1/3 of the cornea [15].

A number of clinical parameters used in this study were defined based on our previous study [25]. The size of the epithelial defect and infiltrate was categorised as very small (<1 mm), small (1–3 mm), moderate (3.1–6 mm) or large (>6 mm), based on the maximum linear dimension. The location of the ulcer was divided into central (any part of the ulcer affecting the visual axis), paracentral (in between the central and peripheral location), and peripheral (the entire ulcer was within 3 mm from the limbus). Recurrence was defined as the re-occurrence of FK after complete resolution of the previous FK episode, irrespective of the time interval between the first and subsequent infective episode. To avoid any duplication of the patient’s risk factors in bilateral or recurrent FK cases, we only included one eye per patient in this study. For recurrent cases, only the first FK episode was included and analysed, regardless of the laterality of infection in the subsequent infective episode.

## 3. Microbiological Culture, Diagnosis and Treatment

Based on the departmental guideline for IK, all patients presented with corneal ulcer(s) of >1 mm diameter, central location or sight-threatening, associated with significant anterior chamber reaction, or atypical presentation were subjected to the microbiological investigation such as corneal scraping for microscopy (with Gram staining), microbiological culture and sensitivity testing [3,5]. Corneal scrapes were inoculated on chocolate agar (for fastidious organisms), blood agar (for bacteria) and Sabouraud dextrose agar (for fungi). For suspected cases of Acanthamoeba keratitis, corneal swab and/or epithelial biopsy was obtained for polymerase chain reaction (PCR) testing [5]. All cultures were incubated for at least 1 week (and up to 3 weeks for suspected Acanthamoeba keratitis). In vivo confocal microscopy (IVCM) using the Heidelberg Retinal Tomography (HRT) II/III with Rostock Cornea Module (Heidelberg Engineering Ltd., Hertfordshire, UK) was utilised to aid the diagnosis or exclusion of fungal and Acanthamoeba keratitis [5].

During the initial treatment phase, all patients with FK were treated with intensive hourly antifungal topical treatment, using either voriconazole 1%, natamycin 5%, amphotericin B 0.15% or a combination of them, based on the severity of infection, types of fungi and clinicians’ preference. Further modification to the treatment regimen and addition of oral antifungal treatment were made according to the patient’s clinical progress and culture results.

### Statistical Analysis

Statistical analysis was performed using SPSS version 27.0 (IBM SPSS Statistics for Windows, Armonk, NY, USA). For descriptive and analytic purposes, the cases were divided into culture-positive and culture-negative FK cases. Comparison between groups was conducted using Pearson’s Chi-square or Fisher’s Exact test where appropriate for categorical variables, and *T*-test or Mann–Whitney U test for continuous variables. Normality of data distribution was assumed if the skewness and kurtosis z-values were between −1.96 and +1.96 and the Shapiro–Wilk test *p*-value was > 0.05. All continuous data were presented as mean ± standard deviation (SD) and/or 95% confidence interval (CI).

The main outcome measures were corrected-distance-visual-acuity (CDVA) and time to complete corneal healing, defined as complete resolution of infection with corneal re-epithelialisation. Snellen vision was converted to logMAR vision for analytic purposes. Counting fingers (CF), hand movement (HM), perception to light (PL) and no perception to light (NPL) were quantified as 1.9 logMAR, 2.3 logMAR, 2.8 logMAR and 3.0 logMAR respectively [25,26]. For patients who underwent keratoplasty (either therapeutic, tectonic or optical), the CDVA immediately prior to keratoplasty was used as the final visual outcome. A final CDVA of 3.0 logMAR was assigned to cases that eventuated with evisceration or enucleation. Logistic regression analysis was performed to examine for any potential prognostic factors for poor visual outcome, defined as corrected-distance-visual-acuity (CDVA) of <1.0 logMAR (or <6/60) and poor corneal healing, defined as >60 days to achieve complete corneal healing from the initial presentation or required tectonic/therapeutic keratoplasty, evisceration or enucleation to resolve the infection. *p*-value of < 0.05 was considered statistically significant.

## 4. Results

### 4.1. Overall Description

During the 10-year study period, 117 patients (*n* = 117 eyes) with FK were included. The mean age was 59.0 ± 19.6 years (range, 4–92 years), 51.3% of patients were female and 57.3% of cases affected the right eye (Table 1). The mean follow-up duration was 26.2 ± 26.5 months. A total of 52 (44.4%) culture-positive FK cases and 65 (55.6%) culture-negative presumed FK cases were included (Table 1). Thirty-two (27.3%) cases were treated as mixed bacterial/fungal keratitis.

### 4.2. Causative Organisms and Risk Factors

*Candida* spp. (33, 62.3%) was shown to be the most common fungal pathogen, followed by *Fusarium* spp. (9, 17.0%) and *Aspergillus* spp. (5, 9.4%; Table 2). Almost all (51, 98.1%) cases were caused by a single fungal pathogen, except for one (1.9%) case which was caused by poly-fungal infection secondary to *Rhodotorula* spp. and *Alternaria* spp. Of all cases, 32 (27.3%) cases were affected by bacterial co-infection, with *Staphylococci spp.* (16, 13.7%) as the most common cause. No significant difference in age (*p* = 0.14), gender (*p* = 0.34) and hospital location (*p* = 0.57) was found between yeast and filamentous FK (Table 3).

All (100%) patients were found to have at least one risk factor, with ocular surface disease (60, 51.3%), prior corneal surgery (44, 37.6%) and systemic immunosuppression (42, 35.9%) as the most common risk factors (Table 1). Ocular surface disease was the most common risk factor for both yeast and filamentous FK (Table 3). Contact lens wear was more commonly associated with filamentous FK than yeast FK (50.0% vs. 18.2%; *p* = 0.017) whereas prior corneal surgery and use of topical corticosteroids were more commonly observed in yeast FK than filamentous FK, though statistical significance was not achieved (both *p* > 0.05).

### 4.3. Clinical Characteristics

The baseline clinical characteristics are summarised in Table 1. The mean interval between the onset of symptoms and the first presentation to the ophthalmic team was 9.5 ± 14.9 days. At baseline, 83 (70.9%) patients presented with a CDVA of <1.0 logMAR. The most frequently observed clinical characteristics of the ulcer were moderate epithelial defect size (45, 38.5%), moderate infiltrate size (47, 40.2%), central location (64, 59.8%) and absence of hypopyon (77, 65.8%). Hospitalisation for intensive treatment was required in 95 (81.2%) patients, with a mean hospitalisation duration of 18.9 ± 16.3 days. Except for presenting CDVA (*p* = 0.038), there was no significant difference in the demographic factors, risk factors and baseline clinical characteristics between culture-positive and culture-negative FK cases (all *p* > 0.05; Table 1). Typical clinical features of FK were present in 93 (79.5%) cases, with feathery border (52, 44.4%), satellite lesions (39, 33.3%) and deep stromal/endothelial plaque (39, 33.3%) being the most common features (Table 4). No significant difference in the typical features was noted between culture-positive and culture-negative cases.

### 4.4. Medical and Surgical Treatment

A total of 51 (43.6%) patients were successfully treated with medical treatment alone, with 66 (56.4%) patients requiring additional surgical interventions for controlling the infection and/or its sequelae. The most common choice of topical antifungal treatment was natamycin (63, 53.8%), voriconazole/other azole drops (57, 48.7%) and amphotericin (51, 43.6%). Adjuvant oral antifungal treatment and intrastromal voriconazole injections were administered in 19 (16.2%) patients and 2 (1.7%) patients, respectively. Emergency therapeutic/tectonic keratoplasty (29, 24.8%) was the most commonly performed surgery, followed by amniotic membrane transplant (18, 15.4%), corneal gluing (17, 14.5%), temporary/permanent tarsorrhaphy (17, 14.5%), evisceration (9, 7.7%), enucleation (2, 1.7%), therapeutic corneal cross-linking (1, 0.9%) and conjunctival hooding (1, 0.9%). Of the 29 tectonic/therapeutic keratopathy, 13 (44.8%) of them failed at the final follow-up (mean duration = 24.3 ± 22.7 months). In addition, 10 (8.5%) patients required elective optical penetrating keratoplasty after the resolution of infection.

### 4.5. Clinical Outcomes and Prognostic Factors

**Figure 2:**
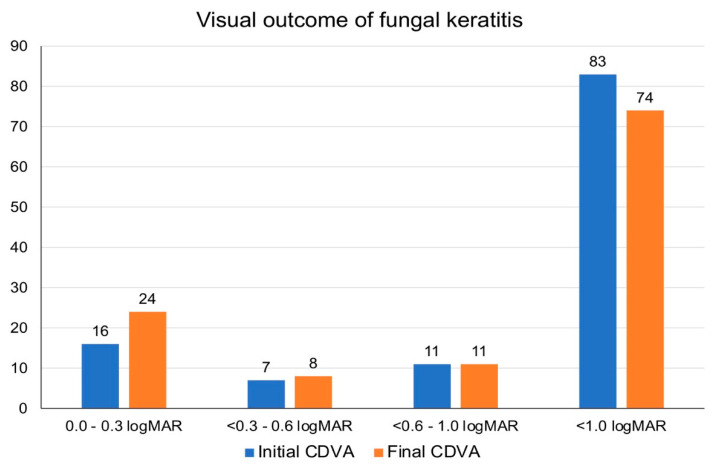
Comparison of corrected-distance-visual-acuity (CDVA) at initial presentation and final follow-up.

The mean CDVA (in logMAR) was similar between initial presentation and final follow-up (1.73 ± 0.90 vs. 1.67 ± 1.08; *p* = 0.36). From the initial presentation to final follow-up, the proportion of patients with CDVA of ≥1.0 logMAR improved from 29.1% to 36.8%, though not statistically significant (*p* = 0.21; Figure 2). Twenty-nine (24.8%) patients had a final CDVA of PL or worse, including 11 (9.4%) patients that eventually required evisceration or enucleation. Nine (7.7%) patients were noted to have a significant cataract but the lens status did not have any significant influence on the visual outcome (therefore it was excluded from the final regression model). Multivariable logistic regression demonstrated that poor visual outcome (CDVA <1.0 logMAR) was significantly influenced by age >50 years old (OR 4.72; 95% CI, 1.40–15.89; *p* = 0.012), presenting CDVA of <1.0 logMAR (OR 14.92; 95% CI, 4.19–53.18; *p* < 0.001) and infiltrate size >3 mm (OR 3.61; 95% CI, 1.11–11.81; *p* = 0.034; Table 5).

A total of 97 (82.9%) patients achieved complete corneal healing at final follow-up, with 11 (9.4%) patients requiring evisceration/enucleation. Seven (6.0%) patients were still undergoing active antifungal treatment at the final follow-up. The mean healing time was 2.71 ± 2.86 months, with 83 (70.9%) patients having a corneal healing time of >60 days. Multivariable logistic regression analysis demonstrated that poor corneal healing was significantly affected by age >50 years (OR 5.81; 95% CI, 1.83–18.37; *p* = 0.003), presenting CDVA of <1.0 logMAR (OR 3.91; 95% CI, 1.19–12.82; *p* = 0.025) and infiltrate size >3 mm (OR 3.91; 95% CI, 1.18–12.88; *p* = 0.025; Table 5). Other factors such as gender, ulcer location, presence of hypopyon, culture results, lens status and co-infection with bacteria did not significantly influence the visual outcome or the corneal healing time (all *p* > 0.05).

### 4.6. Complications

There were various complications noted in this study, including threatened/actual corneal perforation (38, 32.5%), complete loss of vision/NLP (18, 15.4%), new onset of raised intraocular pressure (>21 mmHg)/glaucoma (14, 12.0%), recurrence of infection (12, 10.3%), endophthalmitis (6, 5.1%) and loss of eye (11, 9.4%).

## 5. Discussion

FK is a challenging clinical entity that often results in significant visual impairment and/or blindness. The annual incidence of FK has been estimated to be >1 million worldwide, highlighting the global impact of this disease [10]. Within the UK, several large epidemiological studies have reported a prevalence of 3.0–7.1% of FK among all IK cases [3,27,28,29]. However, there is a lack of literature related to clinical studies on FK in the UK despite its clinical significance and impact. To the best of our knowledge, our study represents the first multi-centre study in the UK that had specifically examined the risk factors, causes and clinical outcomes of FK.

Studies have shown that the causative organisms of FK are influenced by the climates [1,4,10,17,21,30]. Yeast or yeast-like fungi such as *Candida* spp. are more commonly observed in temperate regions whereas filamentous fungi, particularly *Fusarium* spp. and *Aspergillus* spp., normally thrive in tropical climates [1,17]. In this study, we observed that *Candida* spp. accounted for the majority (62.3%) of the culture-positive FK, followed by *Fusarium* spp. (17.0%). This was similar to a previous London study where *Candida* spp. was responsible for 60% of all FK cases, [31]. though a recent London study [24]. observed *Fusarium* spp. (40.5%) as the most common organism of FK, closely followed by *Candida* spp. (38.0%). Khoo et al. [32]. similarly reported *Candida* spp. as the most common (29.2%) fungal isolate for FK in Sydney, which falls in the temperate region. In contrast, the Asian Cornea Society Infectious Keratitis Study (ACSIKS), a large multi-centre study consisting of 8 countries and >6000 patients with infectious keratitis, demonstrated that filamentous fungi such as *Fusarium* spp. (18.3%) and *Aspergillus* spp. (8.3%) were two of the top three organisms of all IK in this region, particularly India and China [4]. Similarly, the Queensland Microbial Keratitis Database demonstrated filamentous fungi as the most common group of fungal pathogens (76.9%) in Queensland, which is mainly a tropical and subtropical region [2].

Risk factors of FK have also been shown to vary considerably among different geographical regions. More importantly, the underlying risk factors have an important contributory role to the causative fungal pathogen. Corneal trauma with the vegetative matter was consistently reported as the most common risk factor of FK in developing countries, particularly those with high agricultural activity [4,17,33,34]. These FK cases were frequently caused by filamentous fungi, namely *Fusarium* spp. and *Aspergillus* spp. [17,33]. This could help explain the low prevalence of filamentous FK in our study where trauma only accounted for 6.0% of all FK cases. In addition, contact lens wear has been shown to be more commonly implicated in filamentous FK than yeast FK, which was demonstrated by our study and other studies [24,30]. On the other hand, yeast infections, particularly *Candida* spp., were commonly observed in eyes with ocular surface diseases, previous history of corneal transplantation and use of topical corticosteroids [24,30,35]. We similarly observed that yeast was more commonly associated with prior corneal surgery and the use of topical corticosteroids, though not statistically significant, likely due to a type II error as a result of low sample size. With the shifting trend in penetrating keratoplasty to lamellar keratoplasty, interface infectious keratitis, including Candida-related infection following endothelial keratoplasty, has become increasingly common in the clinic and poses significant diagnostic and therapeutic challenges [35,36,37]. Therefore, knowledge of the risk factors can provide useful clues to the underlying causative organisms, potentially guiding the choice of antifungal treatment, especially in culture-negative FK cases.

Ocular surface disease (51.3%) and prior corneal surgeries (37.6%) were shown to be the main risk factor of FK in this study. These findings could be attributed to the nature and scope of ophthalmic work provided by the two included study centres, namely the Queen’s Medical Centre, Nottingham and the Queen Victoria Hospital, East Grinstead. Both centres were tertiary ophthalmic referral centres in the UK where complex ocular surface cases such as cicatricial conjunctival diseases, graft-versus-host-disease, limbal stem cell deficiency and neurotrophic keratopathy, amongst others, were being managed, in addition to common conditions such as dry eye disease and infectious keratitis. In addition, there was also a relatively low prevalence of trauma and fewer agricultural activities in the UK, compared to other countries like India and China where trauma is a common risk factor [4,17,33,34].

In this study, 63% of the patients had a final vision of <1.0 logMAR (mean vision = 1.67 ± 1.08). In addition, 25% of the patients required emergency tectonic/therapeutic keratoplasty, highlighting the significant impact of this disease. The poor outcome was similarly observed by Khoo et al. [32]. who reported a median final vision of 1.5 logMAR in patients with FK. In addition, various studies have reported that 25–50% of the patients with FK required additional surgical interventions to resolve the infection, most commonly in the form of therapeutic/tectonic keratoplasty [15,30]. Therapeutic corneal cross-linking (CXL) has also recently emerged as an attractive adjuvant therapy for treating infectious keratitis [38]. although the benefit for FK remained elusive [39,40]. In this study, therapeutic CXL was performed in one patient, which successfully controlled the infection and prevented corneal perforation and the need for emergency keratoplasty. However, it is also important to note that infectious keratitis may also occur following CXL, which has been shown in our study (one patient) and other studies [41,42].

Prajna et al. [43]. previously demonstrated that the visual outcome (at 3 months) was significantly affected by older age, worse presenting visual acuity and larger presenting infiltrate size. In addition, time to complete cornea re-epithelialisation was proportionately correlated with the infiltrate size and increased age whereas larger epithelial defect significantly increased the risk of corneal perforation. Another study by the same group similarly observed the risk of corneal perforation in FK was significantly influenced by the increased size of infiltrate as well as the presence of hypopyon and involvement of 1/3 posterior cornea at presentation [15]. This was similarly observed in our study where increased age and large infiltrate size (>3 mm) served as significant negative predictive factors for visual outcome and corneal healing.

Compared to our recent bacterial keratitis study, [25]. FK was shown to be associated with poorer visual outcomes, a higher need for hospitalisation (with longer duration), longer healing time and higher rate of complications. This was similarly observed in many other studies where FK was shown to fare worse than bacterial keratitis, [4,32,44]. highlighting the significant impact of FK on the patients, healthcare systems and economy (due to loss of work productivity).

Our study represents one of the largest studies in the UK specifically examining the epidemiology, risk factors, causes and outcomes of FK. In addition, we examined the prognostic factors of various clinically important outcomes of FK, including the visual outcome and time to complete corneal healing. The main limitation of this study was the inclusion of culture-negative presumed FK cases. However, we had examined the medical case notes to ensure that all the included cases were true FK cases based on the clinical presentation and clinical course. This was further supported by the similar baseline characteristics of culture-positive and culture-negative FK cases in our study. The issue with low culture yield in infectious keratitis has been a recurrent clinical theme [1,25,45]. In the future, it is envisaged that novel technologies, including polymerase chain reaction (PCR), [46,47], IVCM [48], next-generation sequencing, [49,50,51], matrix-assisted laser/desorption ionisation-time of flight-mass spectrometry (MALDI-TOF-MS) [14,52] and artificial intelligence-assisted platforms [53,54,55,56] would be able to enhance the diagnostic yield and accuracy of infectious keratitis. As contact lenses serve as an important risk factor for FK, future studies examining the influence of the types and brands of CL on the microbiological profiles and risk of infection would be valuable. Comprehensive analysis of antifungal susceptibility of fungal isolates of FK will also be performed in the future to examine the correlation between the susceptibility results and the clinical outcomes.

In conclusion, FK represents an uncommon but challenging ocular pathology that often results in a poor visual outcome, with a high need for surgical interventions. Current therapeutic options are limited in clinical practice. Novel therapies such as host defence peptides (also known as antimicrobial peptides) and phage therapy have demonstrated promise as a potential treatment for treating infectious keratitis and future investigations of these therapy for FK would be valuable [57,58,59,60,61].

## Figures and Tables

**Table 1:** Summary of the demographic factors, risk factors and baseline clinical characteristics of fungal keratitis presented to Queen’s Medical Centre (QMC), Nottingham, UK and Queen Victoria Hospital QVH), East Grinstead, UK.

Parameters	All Cases	Culture-Proven	Culture-Negative	*p*-Value ^#^
	Total N = 117; N (%)	Total N = 52; N (%)	Total N = 65; N (%)	
Hospital				0.12
QVH	87 (74.4)	35 (67.3)	52 (80.0)	
QMC	30 (25.6)	17 (32.7)	13 (20.0)	
Age, years	59.0 ± 19.6	56.5 ± 20.8	61.1 ± 18.5	0.21
Gender				0.11
Female	60 (51.3)	31 (59.6)	29 (44.6)	
Male	57 (48.7)	21 (40.4)	36 (55.4)	
Laterality				0.23
Left	50 (42.7)	19 (36.5)	31 (47.7)	
Right	67 (57.3)	33 (63.5)	34 (52.3)	
Risk factors ^$^				0.37
OSD *	60 (51.3)	30 (57.7)	30 (46.2)	
Prior corneal surgery	44 (37.6)	18 (34.6)	26 (40.0)	
Immunosuppression **	42 (35.9)	21 (40.4)	21 (32.3)	
Contact lens wears	28 (23.9)	17 (32.7)	11 (16.9)	
Topical corticosteroids	19 (16.2)	10 (19.2)	9 (13.8)	
Lid diseases ***	16 (13.7)	4 (7.7)	12 (18.5)	
Trauma	7 (6.0)	3 (5.8)	4 (6.2)	
Presenting CDVA, in logMAR				0.038
0.0–0.3	16 (13.7)	10 (19.3)	6 (9.2)	
<0.3–0.6	7 (6.0)	4 (7.7)	3 (4.6)	
<0.6–1.0	11 (9.4)	8 (15.4)	3 (4.6)	
<1.0	83 (70.9)	30 (57.7)	53 (81.5)	
Size of epithelial defect				0.22
Very small (<1 mm)	6 (5.1)	2 (3.8)	4 (6.2)	
Small (1–3 mm)	38 (32.5)	19 (36.5)	19 (29.2)	
Moderate (3.1–6 mm)	45 (38.5)	23 (44.2)	22 (33.8)	
Large (>6 mm)	28 (23.9)	8 (15.4)	20 (30.8)	
Size of infiltrate				0.52
Very small (<1 mm)	10 (8.5)	5 (9.6)	5 (7.7)	
Small (1–3 mm)	45 (38.5)	21 (40.4)	24 (36.9)	
Moderate (3.1–6 mm)	47 (40.2)	22 (42.3)	25 (38.5)	
Large (>6 mm)	15 (12.8)	4 (7.7)	11 (16.9)	
Location				0.71
Central	70 (59.8)	29 (55.8)	41 (63.1)	
Paracentral	34 (29.0)	17 (32.7)	17 (26.2)	
Peripheral	13 (11.1)	6 (11.5)	7 (10.8)	
Hypopyon				0.76
Yes	40 (34.2)	17 (32.7)	23 (35.4)	
No	77 (65.8)	35 (67.3)	42 (64.6)	
Hospitalisation required				0.29
Yes	95 (81.2)	40 (76.9)	55 (84.6)	
No	22 (18.8)	12 (33.1)	10 (15.4)	
Duration of hospitalisation, days	18.9 ± 16.3	17.5 ± 15.0	19.8 ± 17.2	0.5
Co-infection with bacteria				0.18
Yes	32 (27.3)	11 (21.2)	21 (32.3)	
No	85 (72.7)	41 (78.8)	44 (67.7)	
Need for surgical intervention (s)				0.62
Yes	66 (56.4)	28 (53.8)	38 (58.5)	
No	51 (43.6)	24 (46.2)	27 (41.5)	

OSD = Ocular surface disease; CDVA = Corrected-distance-visual-acuity. Continuous values are presented as mean ± standard deviation (SD). ^$^ Some patients had more than 1 risk factor identified. * Included dry eye disease, meibomian gland disease, neurotrophic keratopathy, previous corneal infection, corneal erosion syndrome, limbal stem cell deficiency, cicatricial conjunctivitis, band keratopathy and bullous keratopathy. ** Included diabetes, use of systemic immunosuppressive drugs, malnutrition and immunodeficiency. *** Included lid ectropion, entropion, distichiasis/trichiasis and exposure keratopathy. ^#^ Comparison between culture-positive and culture-negative cases. Chi-square and unpaired *T*-test were used for categorical and continuous variables, respectively. The significant value is underlined.

**Table 2:** Causative organisms of fungal keratitis and/or co-infection with bacteria that presented to the Queen’s Medical Centre, Nottingham, UK and Queen Victoria Hospital, East Grinstead, UK, between 2011 and 2020.

Organisms	N (%)
Fungi	
Total	53 (100)
Yeast	33 (62.3)
*Candida* spp.	33 (62.3)
Filamentous fungi	19 (35.8)
*Fusarium* spp.	9 (17.0)
*Aspergillus* spp.	5 (9.4)
*Peniophora* spp.	2 (3.8)
*Acremonium* spp.	1 (1.9)
*Scedosporium* spp.	1 (1.9)
Mixed yeast and filamentous infection	1 (1.9)
*Rhodotorula* spp. + *Alternaria* spp.	1 (1.9)
Bacteria (co-infection with fungal keratitis)	
Total *	32 (27.4)
Gram-positive	22 (18.8)
*Staphylococci* spp.	16 (13.7)
*Streptococcus pneumonia*	3 (2.6)
*Bacillus* spp.	2 (1.7)
*Enterococcus faecalis*	1 (0.9)
Gram-negative	10 (8.)
*Moraxella* spp.	3 (2.6)
*Serratia marcescens*	3 (2.6)
*Pseudomonas* spp.	2 (1.7)
*Haemophilus influenza*	1 (0.9)
*Acinetobacter lwoffii*	1 (0.9)

* Percentage calculated based on all the included cases of fungal keratitis (*n* = 117).

**Table 3:** Summary of demographic factors and risk factors based on types of fungal keratitis (FK).

Parameters	Yeast FK	Filamentous FK	*p*-Value ^#^
	Total N = 33;	Total N = 18;	
	N (%)	N (%)	
Age	60.2 ± 18.9	51.3 ± 22.9	0.14
Gender			0.34
Female	21 (63.6)	9 (50.0)	
Male	12 (36.4)	9 (50.0)	
Hospital			0.57
QVH	21 (63.6)	10 (55.6)	
QMC	12 (36.4)	8 (44.4)	
Risk factors ^$^			0.31
OSD	14 (42.4)	11 (61.1)	0.2
Prior corneal surgery	7 (21.2)	2 (11.1)	0.37
Immunosuppression	8 (24.2)	7 (38.9)	0.27
Contact lens wear	6 (18.2)	9 (50.0)	0.017
Topical corticosteroids	6 (18.2)	1 (5.6)	0.21
Trauma	2 (6.1)	1 (5.6)	0.94

A case of poly-fungal keratitis, caused by both yeast and filamentous fungi, was excluded from the analysis. OSD = Ocular surface disease (including lid diseases due to small number). ^#^ Comparison between yeast-like and filamentous FK cases. Chi-square and unpaired *T*-test were used for categorical and continuous variables, respectively. The significant value is underlined. ^$^ Some patients had more than 1 risk factor identified.

**Table 4:** Typical clinical signs of culture-positive and culture-negative fungal keratitis.

Clinical Features	All Cases	Culture-Positive	Culture-Negative	*p*-Value
	Total N = 117;	Total N = 52;	Total N = 65;	
	N (%)	N (%)	N (%)	
Typical clinical signs				0.8
Feathery border	52 (44.4)	25 (48.1)	27 (41.5)	
Satellite lesions	39 (33.3)	16 (30.8)	23 (35.4)	
Deep stromal/endothelial plaque	39 (33.3)	14 (26.9)	25 (38.5)	
Multifocal lesion	32 (27.4)	15 (28.8)	17 (26.2)	
Ring infiltrate	29 (24.8)	13 (25.0)	16 (24.6)	
Number of typical clinical signs				0.74
None	24 (20.5)	12 (23.1)	12 (18.5)	
1	31 (26.5)	15 (28.8)	16 (24.6)	
2	35 (29.9)	13 (25.0)	22 (33.8)	
3 or more	27 (23.1)	12 (23.1)	15 (23.1)	

**Table 5:** Prognostic factors for poor visual outcome [defined as corrected-distance-visual-acuity (CDVA) of <1.0 logMAR]. and poor corneal healing (defined as >60 days to achieve complete healing or required tectonic/therapeutic keratoplasty, evisceration or enucleation) in fungal keratitis in the UK.

	Poor Visual Outcome		Poor Corneal Healing	
Parameters	Odd Ratio (95% CI)	*p*-Value *	Odd Ratio (95% CI)	*p*-Value *
Age > 50 years	4.72 (1.40–15.89)	0.012	5.81 (1.83–18.37)	0.003
Male gender	0.99 (0.33–3.00)	0.99	0.93 (0.33–2.72)	0.91
Right eye	1.13 (0.37–3.43)	0.83	2.86 (0.95–8.61)	0.06
Presenting CDVA < 1.0	14.92 (4.19–53.18)	<0.001	3.91 (1.19–12.82)	0.025
Infiltrate size > 3 mm	3.61 (1.11–11.81)	0.034	3.91 (1.18–12.88)	0.025
Central ulcer	1.51 (0.50–4.53)	0.47	1.58 (0.55–4.58)	0.4
Presence of hypopyon	2.87 (0.81–10.18)	0.1	2.78 (0.78–9.86)	0.12
Culture results		0.88		0.44
Negative	Reference	-	Reference	-
Yeast	1.36 (0.37–4.97)	0.64	1.67 (0.49–5.76)	0.42
Filamentous	0.95 (0.19–4.71)	0.95	2.74 (0.52–14.41)	0.23
Co-infection with bacteria	1.70 (0.49–5.93)	0.4	0.71 (0.23–2.26)	0.57

* Multivariable logistic regression analysis was performed. Significant *p*-values are underlined.

## Data Availability

All relevant data are provided in this study.
